# AI-Enabled Technologies and Biomarker Analysis for the Early Identification of Autism and Related Neurodevelopmental Disorders

**DOI:** 10.3390/children12121670

**Published:** 2025-12-09

**Authors:** Rohan Patel, Beth A. Jerskey, Jennifer Shannon, Neelkamal Soares, Jason M. Fogler

**Affiliations:** 1 Department of Pediatrics, University of North Carolina at Chapel Hill, 1828 MLK Jr Blvd, Chapel Hill, NC 27544, USA; rohan.patel@unc.edu; 2 Boston Child Study Center, 729 Boylston Street, Fifth Floor, Boston, MA 02116, USA; bjerskey@bostonchildstudycenter.com; 3 Department of Psychiatry and Human Behavior, Warren Alpert Medical School of Brown University, 22 Richmond Street, Providence, RI 02903, USA; 4 Psyched About AI, Inc., 15600 Ne 8th Street Ste B1, #267, Bellevue, WA 98008, USA; psych@jennifershannonmd.com; 5 Department of Pediatrics, University of Michigan Medical School, 1522 Simpson Rd East, Ann Arbor, MI 48109, USA; soaresne@med.umich.edu; 6 Division of Developmental Medicine, Department of Pediatrics, Boston Children’s Hospital, 2 Brookline Place, 4th Floor, Brookline, MA 02445, USA; 7 Departments of Pediatrics and Psychiatry, Harvard Medical School, 25 Shattuck St, Boston, MA 02115, USA

**Keywords:** autism spectrum disorder, artificial intelligence, machine learning, early detection, digital biomarkers, eye-tracking, neurodevelopmental disorders

## Abstract

**Highlights:**

**What are the main findings?**

- AI-enabled tools such as eye-tracking, acoustic analysis, and video-based classifiers demonstrate high diagnostic accuracy and specificity in identifying autism spectrum disorder (ASD), often rivaling traditional observational methods.
- Multimodal integration of behavioral, physiological, and clinical data significantly enhances predictive power compared to single-modality approaches.

**What are the implications of the main findings?**

- These technologies offer scalable solutions that can reduce wait times and specialist bottlenecks, enabling earlier ASD identification in primary care and home settings.
- Ethical adoption requires addressing algorithmic bias, ensuring data privacy, and establishing clear regulatory and reimbursement pathways to support equitable clinical deployment.

**Abstract:**

**Background:** Autism spectrum disorder (ASD) and related neurodevelopmental conditions are a significant public health concern, with diagnostic delays hindering timely intervention. Traditional assessments often lead to waiting times exceeding a year. Advances in artificial intelligence (AI) and biomarker-based screening offer objective, efficient alternatives for early identification. **Objective:** This review synthesizes the latest evidence for AI-enabled technologies aimed at improving early ASD identification. Modalities covered include eye-tracking, acoustic analysis, video- and sensor-based behavioral screening, neuroimaging, molecular/genetic assays, electronic health record prediction, and home-based digital applications or apps. This manuscript critically evaluates their diagnostic accuracy, clinical feasibility, scalability, and implementation hurdles, while highlighting regulatory and ethical considerations. **Findings:** Across modalities, machine learning approaches demonstrate strong accuracy and specificity in ASD detection. Eye-tracking and voice-acoustic classifiers reliably differentiate for autistic children, while home-video analysis and Electronic Health Record (EHR)-based algorithms show promise for scalable screening. Multimodal integration significantly enhances predictive power. Several tools have received Food and Drug Administration clearance, signaling momentum for wider clinical deployment. Issues persist regarding equity, data privacy, algorithmic bias, and real-world performance. **Conclusions:** AI-enabled screeners and diagnostic aids have the potential to transform ASD detection and access to early intervention. Integrating these technologies into clinical workflows must safeguard equity, privacy, and clinician oversight. Ongoing longitudinal research and robust regulatory frameworks are essential to ensure these advances benefit diverse populations and deliver meaningful outcomes for children and families.

## 1. Introduction

Autism spectrum disorder (ASD) refers to a group of neurodevelopmental disorders that develop in early childhood and are characterized by challenges in social communication and interaction as well as restricted, repetitive behaviors or interests [1]. Recent surveillance shows prevalence rates in the United States (U.S.) of approximately 1 in 31 children (3.2%), with a male-to-female ratio of about 3:1 [2].

Although clinical features of ASD are well described, individual presentation varies widely, behaviorally, and neurodevelopmentally, due to multiple contributing factors [3]. Social difficulties may manifest such as poor eye contact, reduced gestures, limited conversational reciprocity, and diminished awareness of self or others’ emotions. Language abilities can range from nonverbal or minimal speech to hyperlexic speech that might be highly repetitive, echolalic, or unusually intonated. Behaviors may involve rigidity, repetitive mannerisms, intense interests, and/or atypical sensory seeking or avoidance.

ASD is considered multifactorial. While certain genetic and environmental risk factors are recognized, the etiology is not yet fully understood. Approximately 10–25% of children with ASD have a known genetic or chromosomal disorder, especially with broad genomic testing. However, less than 1% of non-syndromic ASD cases stem from mutations in any single gene; large-scale sequencing studies implicate both rare variants and common polygenic risk [4].

Identified risk factors for ASD include family history (e.g., affected siblings), prematurity, and advanced parental age. Myths such as vaccine causation have been disproven [5].

Diagnosis is currently based on a child’s developmental history and behavior, assessed through caregiver interviews, standardized reports, and direct semi-structured observation. Most early signs emerge by age two years, including reduced social engagement and communication delays. Despite the possibility of diagnosing ASD as early as 18 months, surveillance data show that the median age of earliest known ASD diagnosis in the U.S. is about 47 months, with substantial variability across states (e.g., 36 months in California and 69.5 months in parts of Texas) [6]. No single symptom or sign is pathognomonic, and diagnostic accuracy relies on gathering symptom details to distinguish ASD from overlapping neurodevelopmental or neuropsychiatric conditions.

### 1.1. Public-Health Challenge and Current Diagnostic Standards

Initiating intervention as early as possible yields the greatest gains for children on the autism spectrum. Children identified and treated earlier are more likely to attend mainstream school and show superior verbal and cognitive outcomes compared with those diagnosed later [7]. Specifically, a community-treated cohort of 131 children diagnosed with ASD between 1.2 and 5 years found that those diagnosed before 2.5 years were three times more likely to show substantial improvements in core social symptoms over 1–2 years [8]. These findings bolster the case for universal ASD screening before 2.5 years. Even a modest timing difference matters: Starting treatment at 18 months versus 27 months was associated with better outcomes [9], and earlier initiation of ABA relates to greater improvements [9]. Furthermore, high-quality early intervention is associated with a reduced need for long-term educational services and a significant cost-offset [10,11].

Despite the compelling case for early diagnosis and treatment, the current process in the U.S. involves referral from primary care physicians (PCPs) to specialist clinicians who often have waiting lists extending over months or years. A 2025 survey of U.S. autism specialty centers reported that nearly two-thirds had wait times longer than four months, and about 15% experienced waits exceeding a year or had closed waitlists [12].

These bottlenecks stem in part from documentation-intensive workflows [13], a “one-size-fits-all” approach to assessment when a tiered approach would be more efficient [14], and ongoing clinician shortages. Surveys of U.S. developmental-behavioral pediatricians report that excessive documentation not only reduces direct patient interaction but also increases provider burnout [13]. These factors combine to delay the identification of neurodevelopmental disorders, especially in under-resourced regions.

To address this, the recent literature and American Academy of Pediatrics (AAP) guidance suggest that autism diagnosis need not follow a rigid, one-size-fits-all model [15]. In straightforward cases, PCPs—when equipped with appropriate tools and training—can make accurate diagnoses without requiring multidisciplinary, multi-day evaluations [14,15]. A statewide study demonstrated that PCPs achieved over 80% diagnostic agreement with specialists for young children referred with developmental concerns [14,15]. This supports a tiered diagnostic approach, where assessment intensity is matched to case complexity. While promising, this model has yet to gain widespread traction due to reimbursement challenges, variability in training, and limited infrastructure.

### 1.2. Emergence of AI-Enabled Screening and Diagnostics

Given these persistent challenges researchers and clinicians are exploring how artificial intelligence (AI) could offer rapid, accurate screening while supporting objective analysis by combining data from multiple modalities. Unlike traditional methods, which rely heavily on subjective observation and can be delayed due to lack of clinician availability, AI-enabled tools can process vast datasets to identify subtle neurodevelopmental patterns—such as micro-movements or acoustic inflections—that may escape the human eye. This capability not only enhances diagnostic accuracy by reducing observer bias but also significantly improves timeliness, potentially enabling identification within the critical neuroplasticity window before age three. Initial objective assessment methods, such as genetic assays and neuroimaging, often proved infeasible for widespread, routine use due to prohibitive costs, the possible need for sedation (for neuroimaging), and insufficient diagnostic performance or reproducibility [16].

AI tools now allow for the quantification of subtle behavioral and physiological markers that may elude subjective observation. Machine learning (ML) algorithms can analyze multimodal data (such as eye movements, voice patterns, physical activity, neuroimaging, and electronic health records [EHRs]) to identify patterns associated with ASD before clinical symptoms are overt [17,18]. Automating feature extraction and pattern recognition, these tools enable scalable early screening and diagnosis in medical homes like primary care and home settings, which would otherwise face significant difficulties, and simultaneously improve the efficiency and comprehensiveness of specialty practices.

### 1.3. Scope of This Review

This review offers a clinically focused synthesis of current research on AI-enabled solutions designed to overcome diagnostic barriers in ASD. We conducted a narrative review of the literature, prioritizing articles from major medical databases (including PubMed) that report on validated or emerging technologies. The final selection of studies reflects expert consensus on the most promising tools for reducing diagnostic bottlenecks. Recent advancements in AI-enabled technologies for early identification of ASD and other neurodevelopmental disorders show promise. We begin by reviewing core AI concepts, specifically predictive modeling, and natural language processing (NLP), within the diagnostic context (Section 2). The primary body of evidence, structured by distinct modalities, is presented in Section 3. Following this, we critically evaluate diagnostic performance and methodological rigor (Section 4), examine regulatory and ethical considerations (Section 5 and Section 6), and finally with future directions (Section 7) and conclusions (Section 8).

Specifically, this narrative review examines the screening and diagnostic accuracy, clinical feasibility, and real-world implementation barriers for the following technologies:

- ML analysis of behavioral videos and caregiver surveys
- NLP of clinical notes and developmental histories
- Computer vision applications for eye-tracking and facial expression
- Acoustic analysis of vocalizations and speech
- Wearable sensor data for movement and physiology
- Hair analysis for metabolomic biomarkers
- Neuroimaging-derived markers
- EHR-based risk prediction and data integration
- Home-based digital screening applications (apps)

## 2. Foundational AI Technologies in Neurodevelopmental Diagnostics

### 2.1. ML as the Engine of Predictive Diagnostics

ML uses computational algorithms to detect patterns in clinical and behavioral data, allowing early identification of ASD risk. For example, a large recent study of more than a million children demonstrated that ML models combining demographics and developmental milestones can identify 45% of ASD cases—while achieving 95% specificity, meaning most flagged as “not autism” truly are not [18]. Such models, using readily available clinic data, could alert providers to risk years before traditional referral pathways.

Speech and video analysis offer additional, scalable approaches. One study used ML to analyze children’s voice patterns during a repetition task, accurately distinguishing ASD from neurotypical status (91% accuracy) and from other language delays (85% accuracy) [19]. In video-based screening, home videos submitted by families enabled models to classify ASD status with remarkable sensitivity (90–99%)—but specificity was more variable, ranging from 58% to 97% depending on case mix and video quality [20]. Follow-up work using smartphone video collected in global, low-resource settings confirmed that mobile-based video analysis can expand access and maintain high detection rates, but balancing sensitivity and specificity remains a key challenge [21]. This wide range suggests these tools are extremely sensitive for detecting ASD but may flag non-autistic children as at-risk more often in some populations; model calibration and diverse real-world validation are needed.

Other ML studies examine high-dimensional patterns to identify unique ASD subtypes—by clustering children’s gaze, motor or genetic profiles; these approaches may one day support more personalized intervention [22]. Foundational work also evaluated a variety of sensing and robotic technologies in early screening [23]. Consistently, integrating multiple data streams—combining eye tracking, audio, and standardized reports—yields more reliable risk estimates than any single modality alone [24].

Unlike traditional methods, which rely heavily on subjective observation and can be delayed by clinician availability, AI-enabled tools process vast datasets to identify subtle neurodevelopmental patterns. This capability not only enhances diagnostic accuracy by reducing observer bias but also significantly improves timeliness, potentially enabling identification within the critical neuroplasticity window before age three.

Regarding operational feasibility, it is important to distinguish between the development of these models and their clinical application. While the underlying data analysis involves complex high-dimensional processing performed by data scientists, the clinical interface is typically designed for ease of use. For example, in the Earlipoint Evaluation System, the analysis of inputs of eye-tracking is automated: the clinician or technician captures the data (eye-tracking), and the system generates a score or probability report within minutes. Consequently, while specialized training is required to *administer* the test and ensure data quality, the *interpretation* of the AI output does not require the clinician to be a machine learning expert, though they must understand the tool’s scope and limitations.

### 2.2. Natural Language Processing and Speech Analysis

Natural Language Processing (NLP) is an ML technique that allows computers to analyze or generate human language. In developmental diagnostics, NLP can transcribe and interpret caregiver–child conversations or clinician notes to identify early signs of language or social differences.

Beyond basic speech analysis, recent studies show that models can distinguish subtle differences in infant cries—offering a scalable tool for early ASD screening [19]. Other systems convert features like tone, pitch, and rhythm into numerical values, allowing ML algorithms to classify autistic and non-autistic speech with high accuracy.

NLP also helps unlock value from electronic health records, flagging unstructured comments or referral notes that may signal ASD risk when combined with structured data. As generative AI evolves, producing and summarizing enormous amounts of clinical text may support even earlier detection—provided there are safeguards against incorrect or fabricated output (colloquially referred to as “hallucinations”) [25].

## 3. Targeted Review of AI-Enabled Screening and Diagnostic Advances

Section 3 reviews diagnostic accuracy, feasibility, and limitations across several prominent AI-enabled modalities for early ASD identification. Key metrics include sensitivity (correctly identifying children with ASD), specificity (correctly identifying those without), positive predictive value (how likely a positive result truly represents ASD), and negative predictive value (how likely a negative result truly represents non-ASD). Table 1 summarizes major tools, their performance, and main trade-offs, specifying which are validated as screeners or are Food and Drug Administration (FDA)-cleared diagnostic aids.

### 3.1. Eye-Tracking Technologies

Eye-tracking measures where children look; such as the proportion of time spent gazing at faces versus objects—while they view brief social and non-social movies. EarliPoint is an FDA-cleared eye-tracking system that objectively measures a toddler’s social attention patterns—specifically, the proportion of time spent gazing at faces versus objects during brief movies, differentiating autistic and neurotypical toddlers in a single twelve-minute session [26]. A phase III prospective trial reported a sensitivity of 78%, specificity of 85.4%, accuracy of 82.1% and an AUROC of 0.90 when compared with expert clinical diagnosis [26]. These devices are objective and scalable but require dedicated hardware, controlled lighting, and trained staff. Ongoing research seeks to extend eye-tracking to more naturalistic or home-based settings, yet technical challenges such as calibration, motion artifacts and attention to stimuli remain substantial.

### 3.2. Acoustic and Speech-Pattern Analysis

ML programs can analyze vocal patterns and speech features during brief interactions. One study that extracted acoustic features from a non-word repetition task reported 91% classification accuracy for distinguishing autistic from typically developing children and 85% accuracy when the comparison group included children with heterogeneous non-autistic speech and language delays [19]. Deep learning methods for infant cry analysis also show promise as early, scalable risk indicators [27,28]. However, these models require verbal output, are generally evaluated in school-aged children, and need validation across languages, ages, and clinical contexts.

### 3.3. Behavioral Biomarkers from Video and Sensor Data

Computer vision can detect ASD-specific movement, gaze, expression, and motor patterns from short home videos. In a multiclassifier study of home videos, the top logistic-regression model achieved an accuracy of 88.9%, sensitivity of 94.5% and specificity of 77.4% [20]. All classifiers-maintained sensitivity above 94.5%, but specificity ranged widely; specificity was highest (90%) when restricting the cohort to children aged 4–6 years [21]. Performance can match specialist assessment teams, yet results depend heavily on video quality, case mix, and participation. Practical issues like caregiver compliance, privacy and potential false positives require additional study [20,21].

### 3.4. Neuroimaging-Based Machine-Learning Application

Prior to 2022, significant challenges such as small datasets, limited computing resources, and high feature dimensionality hampered generalizability and slowed clinical translation [29]. Recently, AI applied to neuroimaging has shown increasing promise for early ASD detection, particularly in the last two years. While early studies using fMRI and EEG with ML reported moderate to high accuracy (70–95%) [30,31], these models were typically limited to small, homogeneous samples and lacked longitudinal utility, especially compared to more accessible tools like behavioral or home-based screening.

In the last two years, improvements in computational infrastructure, increased access to large open datasets and the rise in multimodal deep-learning architectures have driven renewed momentum. Newer models that combine resting-state fMRI, diffusion tensor imaging, structural MRI, and EEG have demonstrated improved performance and are beginning to predict neurodevelopmental trajectories rather than providing only binary classification [29]. Emerging multimodal neuroimaging reviews highlight the integration of imaging data with AI-driven biomarkers and call for longitudinal research to validate these approaches [32,33]. Neuroimaging-genetic approaches linking atypical brain connectivity with ASD-related genes may yield risk biomarkers with higher specificity [34]. Still, child compliance, interpretability and cost remain significant barriers. Surveys of ML applications in neuroimaging and neural modulation also emphasize the need for large, diverse datasets and transparent methodologies [35,36,37]. Collectively, these multimodal, AI-enabled neuroimaging tools are now positioned as valuable additions to the ASD biomarker landscape and warrant broader inclusion in screening discussions.

### 3.5. AI-Enhanced Molecular and Genetic Assays

Advances in ML and high-throughput genomic technologies have accelerated the search for molecular and genetic biomarkers relevant to ASD diagnosis. Using multi-omics data and feature selection methods, researchers have identified risk-associated gene expression patterns and epigenetic changes in children with ASD. For example, large blood-based transcriptome analyses can distinguish children with and without ASD, although results reveal substantial heterogeneity across populations [38,39]. ML models have also been applied to integrate genetic and environmental risk factors, supporting the view that both rare mutations and environmental exposures influence individual diagnoses [40,41]. While emerging studies combining transcriptome, epigenome and metabolomic data show improvements in early risk prediction—especially when using advanced ML architectures none of these approaches yet have sufficient reproducibility or specificity to support standalone clinical practice. Ongoing research is focused on validation across diverse cohorts, addressing confounders like sample source, and establishing regulatory and analytic standards.

### 3.6. Hair-Based Metabolomic Biomarkers

Recent studies highlight that hair analysis offers a promising, non-invasive biomarker source for early ASD detection, relying on ML to analyze elemental and metabolomic signatures. The ClearStrand-ASD assay developed by LinusBio analyzes elemental and metabolomic signatures across a single strand of hair to build longitudinal profiles of exposure and metabolic fluctuations [42]. In a multi-center study of more than 1500 children, the test demonstrated sensitivity around 81% and a negative predictive value (NPV) around 92% for ruling out ASD [43]. An independent report summarizing an interim analysis of high-risk children reported a similar negative predictive value and noted that the assay provides results in about three weeks. Because of its high NPV, ClearStrand-ASD is marketed as a rule-out test to aid clinicians rather than a stand-alone diagnostic [41]. While dynamic, longitudinal metabolic profiling of hair may prove valuable, independent validation is limited and the technology is still early in its clinical adoption. Ongoing studies are required to confirm accuracy, determine generalizability across diverse populations and environments, and explore integration within broader ASD surveillance systems.

### 3.7. EHR-Based Risk-Prediction Algorithms

Large-scale electronic health records can identify subtle patterns to enhance developmental surveillance. In a cohort of 1.18 million children, an ML model that used longitudinal developmental milestones plus minimal demographics yielded an AUROC of 0.83 and achieved 45.1% sensitivity at 95% specificity [18], indicating modest ability to flag high-risk infants while minimizing false positives. A separate model trained on a minimal set of medical and background variables produced an AUROC of 0.895 with sensitivity 0.805 and specificity 0.829 and validated across independent cohorts with an AUROC of 0.790 [44]. These risk scores can be embedded in well-child EHR systems to trigger early referral or targeted screening (e.g., eye-tracking or video analysis). However, prediction performance depends on data quality and completeness; under-represented populations with limited healthcare access may not be captured and a sizable proportion of children flagged as elevated risk will not meet ASD criteria. Incorporating parent-reported concerns and social determinants of health may improve sensitivity.

### 3.8. Home-Based Digital Screening Applications

SenseToKnow, a digital app developed by Duke University with National Institutes of Health (NIH) funding, facilitates remote phenotyping for caregivers by utilizing a tablet to display short movies and record gaze, facial expressions, and motor responses via the front-facing camera [24]. A multi-clinic study involving 475 toddlers (17–36 months) demonstrated the app’s effectiveness, achieving 87.8% sensitivity and 80.8% specificity, with consistent performance across different sexes and racial groups [24]. When SenseToKnow assessments were combined with the Modified Checklist for Autism in Toddlers (M-CHAT), the positive predictive value significantly increased. The app’s flexibility allows for administration during routine well-child visits or at home, which could help reduce healthcare disparities in rural or underserved regions [24]. A key advantage of AI-driven home apps over conventional parent-report instruments (like the M-CHAT) is the reduction in observer bias. While traditional screeners rely on caregivers’ literacy and subjective interpretation of behavior, computer vision tools objectively measure physiological and motor responses. Currently, most home-based apps function as risk assessments (screeners) rather than diagnostic tools; they do not provide a definitive diagnosis but rather a probability score to prioritize referrals. They typically do not require trained professionals for administration, leveraging the ubiquity of smartphones, though professional interpretation of the results is recommended. However, the app has limitations, including its reliance on caregiver compliance, internet access, and the need for training to accurately interpret results, and it has not yet received regulatory clearance.

## 4. Critical Evaluation of Evidence and Methodologies

### 4.1. Diagnostic Accuracy and Validation Across Populations

The accuracy of AI-enabled tools varies significantly due to differences in study design, sample size, and population diversity. The regulatory landscape shows progress with the FDA clearance of EarliPoint (2022) and authorization of Canvas Dx (2021, via DeNovo pathway). These tools demonstrate reliable metrics through multicenter prospective validation.

EarliPoint’s phase III trial reported sensitivity of 78%, specificity of 85.4% and overall accuracy of 82.1% [26], values that align with post-market observations. Canvas Dx combines a clinician-administered questionnaire caregiver questionnaires, and short home videos, that provides an output of positive for autism, negative for autism or indeterminate when it can not determine with high accuracy among toddlers aged 18–72 months with concern for developmental delay. In a multi-site trial, it demonstrated high predictive value for ASD classification in children who received a determinate output (PPV 80.8%, NPV 98.3%) [45]. In a real-world study of 124 children, Canvas Dx delivered a positive predictive value of 94.4% and a negative predictive value of 95.2%, with determinate results generated for 60.5% of cases [46].

Crucially, most validation studies to date have been cross-sectional. With the exception of select longitudinal cohorts in hair analysis [43] and preliminary neuroimaging trajectory studies [29], few investigations have conducted longitudinal follow-up to confirm that children flagged by AI tools retained their ASD diagnosis at school age.

In contrast, many proof-of-concept studies in acoustic or video domains often involve small, homogeneous samples (typically a few dozen children), which raise concern about overfitting and limited generalizability. Validation across diverse populations, particularly with respect to race, ethnicity, language, and socioeconomic status is essential. The SenseToKnow trial reported consistent accuracy across sex and racial groups [24], but the sample was still predominantly drawn from clinical settings. EHR-based models developed in high-resource healthcare systems may not perform similarly in low-resource contexts. Without intentional inclusion of bilingual and rural populations, AI tools risk exacerbating existing disparities. Future studies should adopt stratified sampling and report subgroup performance to ensure equitable diagnostic accuracy.

### 4.2. Integration of Multi-Modal Data Streams

One of ML’s key strengths lies in its ability to integrate diverse data types, which significantly accelerates the process of identifying patterns and enhances diagnostic accuracy and risk prediction. A prime illustration is Canvas Dx, a tool that combines a caregiver questionnaire, a clinician-administered questionnaire, and two short home videos. Its internal validation demonstrated that this multimodal algorithm consistently outperformed any single component [46]. Similarly, research models that merge eye-tracking data, audio analysis, and parent-reported developmental milestones show improved predictive power compared to single-modality approaches [24]. This multimodal integration captures complementary signals for instance, a child exhibiting atypical gaze patterns, but typical vocalizations might still raise concerns when these subtle cues are combined with other indicators [24].

However, the inherent complexity of integrating these varied data streams presents challenges for model interpretability and regulatory approval. Understanding why an AI model flags a child as elevated risk can be difficult if it is based on a complex interplay of hundreds of data points from eye-tracking, audio, and video [24]. Similarly, gaining regulatory approval for a device that combines multiple data sources, each with its own set of validation requirements, can be a lengthy and intricate process. Transparent architecture and explainable-AI methods are crucial, enabling clinicians to understand the specific features that drive risk predictions [47]. These technologies not only accelerate data capture and synthesis but, when correctly trained, can also identify patterns that human-driven methods might miss, thereby improving prediction and decreasing the time to diagnosis and treatment [19,24].

Ultimately, the role of the clinician remains central. In this AI-assisted model, the algorithm functions as a high-precision data gatherer, surfacing probabilities and flagging subtle biomarkers. The clinician’s responsibility shifts from raw data observation to synthesizing these AI findings with the child’s medical history, family context, and differential diagnoses. The AI provides the ‘what’ (the risk score), while the clinician provides the ‘why’ and manages the ‘what next’ (intervention planning).

### 4.3. Real-World Feasibility and Limitations

Feasibility varies across modalities. Eye-tracking systems require specialized hardware and controlled calibration; for instance, a busy primary care clinic might struggle to dedicate a separate room and trained staff for the 12 min EarliPoint assessment. Video-analysis tools can be implemented via smartphone, but reliance on high-quality recordings and caregiver compliance is non-trivial; for example, a parent might find it challenging to consistently capture clear video of their child’s behavior. Acoustic analysis depends on the child’s ability to produce sufficient speech; thus, it is less applicable in preverbal toddlers. Neuroimaging remains impractical for routine screening due to cost and the possible need for sedation. EHR-based risk models are scalable but have moderate sensitivity; they should trigger, not replace, further assessment. Home-based apps like SenseToKnow show promise for remote screening, but integration into clinical workflows and reimbursement pathways remain unresolved, meaning clinics lack clear guidance on how to incorporate these into their existing systems or get paid for using them.

**Additional clinical and phenotypic limitations:**

- **Phenotypic breadth:** Current AI tools predominantly focus on social-communication and repetitive behaviors. There is a need for models that address other early atypical signs, specifically feeding difficulties (e.g., food neophobia), sensory processing abnormalities (tactile, oral, olfactory), and oromotor deficits, which are often prodromal markers of ASD.
- **Longitudinal validation:** While diagnostic accuracy is high, few studies have conducted long-term follow-up to validate AI screening outcomes against professional clinical assessments years later. Furthermore, there is a lack of data comparing AI-based risk findings with post-rehabilitation results to assess the tool’s long-term predictive value and utility in outcome prediction.
- **Role in rehabilitation:** While this review focuses on identification, AI also holds potential in rehabilitation. Future tools may track micro-progressions in therapy that standard reports miss, though this application remains nascent.

## 5. Regulatory Snapshot and Reimbursement

To date, AI-enabled diagnostic technologies for ASD represent a significant leap forward in addressing the challenges of early identification. These tools offer the potential for more objective, efficient, and scalable screening and diagnosis, often rivaling or even exceeding the accuracy of traditional methods. As evidenced by the FDA clearance of devices like EarliPoint and FDA authorization of Canvas Dx [48,49] (see Table 1), there is growing regulatory acceptance and momentum for their clinical deployment. These technologies accelerate data capture and synthesis, and when correctly trained, can identify subtle patterns that human-driven methods might miss, the time to diagnosis and intervention. However, their real-world feasibility is influenced by factors such as specialized hardware requirements, the need for high-quality data input, and the ongoing challenge of seamless integration into existing clinical workflows and reimbursement structures.

Tools must comply with data privacy standards (e.g., Health Insurance Portability and Accountability Act of 1996 or HIPAA), laboratory regulations (e.g., Clinical Laboratory Improvement Amendments (CLIA) for biomarker assays), and institutional protocols for research and clinical use. Health systems may require internal validation, payer alignment, and EHR integration before widespread adoption. These layers of oversight influence not only safety and efficacy but also scalability, equity, and sustainability in real-world settings.

Current procedural terminology (CPT) codes 96,110 (developmental screening) and 96,112 (developmental testing) do not explicitly cover AI-enabled diagnostic tests. Consequently, reimbursement for devices like EarliPoint or apps like SenseToKnow remains uncertain. Some others, like ClearStrand ASD, are CLIA-approved lab tests and can be considered as such, with no direct reimbursement to the ordering physician. Pilot implementation may proceed through research grants or institutional support, but widespread adoption will require updated coding structures that recognize digital behavioral biomarkers.

## 6. Ethical and Implementation Considerations

Ethical considerations are critical in the adoption of AI for developmental diagnostics. Relevant guiding principles include patient autonomy, informed consent, privacy, validity, and equity of access. As these tools are integrated into clinical workflows, it is essential to consider the implications for justice, fairness, and the obligation to ‘do no harm.’ The ethical framework should ensure that AI complements, rather than replaces, professional judgment while addressing potential for bias and health disparities. Given that the most current American Psychological Association (APA) Ethical Principles of Psychologists and Code of Conduct (also known as the APA Ethics Code) was last updated in 2016 [50], it does not specifically outline the use of AI. However, there are many instances in the code that apply indirectly. Specifically, the APA Ethics Code implies that psychologists using AI must: provide informed consent and disclose limitations (Standard 3.10), protect privacy and data security (Standard 4.01), remain competent and educated about the tools (Standard 2.01) and ensure scientific validity before use (Standards 9.01, 2.04).

Health systems should develop “pilot-to-scale” roadmaps: begin with subspecialty clinics to build clinician familiarity, collect post-market data, and gradually expand to primary care once workflows are optimized. Alignment with health-information standards (e.g., Health Level 7 Fast Healthcare Interoperability Resources (FHIR)) will facilitate integration into electronic health records and decision-support systems.

### 6.1. Algorithmic Bias and Equity

APA Ethics Code Principal D (Justice) applies here as supporting equal quality in the processes, procedures, and services being conducted. In addition, APA Ethics Code Standard 9.06: Interpreting Assessment Results suggests that “When interpreting assessment results, including automated interpretations, psychologists take into account the purpose of the assessment.” Although drafted to likely reflect interpretations of well validated tests like the Minnesota Multiphasic Personality Inventory [MMPI] [51], this standard could be expanded to include interpretations derived from biomarkers. Like well-known psychological assessments, AI-derived interpretations should not be used in place of clinical judgment.

AI models are only as fair as the data on which they are trained. Many training datasets underrepresent bilingual children, racial and ethnic minorities, and rural families. Tools developed using homogeneous samples can yield false negatives in under-represented groups, reinforcing disparities in early diagnosis. The SenseToKnow team explicitly evaluated performance across sex and racial groups and observed consistent accuracy [23], but such analysis is rarely reported elsewhere. Developers must quantify and report dataset diversity, adopt bias-mitigation techniques (e.g., re-weighting, adversarial debiasing), and validate models in diverse populations. For eye-tracking, calibrations that assume uniform ocular morphology may inadvertently penalize children with nystagmus or strabismus; alternative stimuli or dynamic calibration may improve inclusivity.

- **Validity and reliability**: AI must be rigorously validated including sensitivity, specificity, and predictive values across diverse populations and settings before clinical use. Unvalidated tools risk harm from wrong diagnoses.
- **False positives/false negatives** (harms of misclassification): False positives can cause anxiety, stigma, unnecessary interventions and resource diversion, while false negatives delay needed services. Both outcomes have real developmental and psychosocial consequences. There must be a balance between the two deciding whether it is better to have more false positives to get screened (and then seen) versus false negatives that delay treatment and diagnosis.
- **Bias**: A truly objective system must also be equitably designed and validated across diverse demographic and linguistic backgrounds. Health literacy differences can affect participation and opt-out rates, potentially biasing the underlying data and subsequent model performance.

### 6.2. Data Privacy and Consent

In addition to cautions around consent, Standard 4.01 Maintaining Confidentiality suggests that psychologists have a primary obligation and take reasonable precautions to “protect confidential information obtained through or stored in any medium, recognizing that the extent and limits of confidentiality may be regulated by law or established by institutional rules or professional or scientific relationships.” Parents/caregivers (and when appropriate older children/adolescents) should not only be informed about what (and how) data will be collected, but also the limits of accuracy and options if the algorithm flags a concern. In addition, AI-enabled diagnostics rely on sensitive data (e.g., high-resolution videos) and for pediatric cohorts, obtaining informed parental consent and ensuring data security are paramount. Data sharing between healthcare providers and technology companies raises questions about ownership and control; parents must understand how their child’s data will be used and stored, who will have access to it, and how it will be retained. Compliance with HIPAA (United States) and General Data Protection Regulation (GDPR) (Europe) regulations is essential, yet cross-border data transfer may complicate compliance. Institutions should adopt transparent data-governance frameworks and provide opt-out options for families.

### 6.3. Trust and Explainability

APA Ethics Code Principal B (Fidelity and Responsibility) describes how psychologists are expected to establish relationships of trust with patients and the public. It emphasizes honesty, accountability, and avoiding conflicts of interest. Clinicians, families, and regulators need a clear understanding of how an AI model works, what inputs it uses, its limitations, and why it produced a particular result. Parents and clinicians may be wary of “black-box” algorithms. Explainable-AI (ExAI) techniques such as gaze heat-maps that visualize areas of interest or feature-importance rankings for speech classifiers can build trust by showing how the model arrived at its conclusion [47]. For example, an eye-tracking report could display the proportion of time spent looking at eyes versus objects, enabling parents to relate the result to observable behavior. Despite the theoretical benefits, widespread adoption of transparent, explainable AI systems is limited by technical complexity, regulatory hurdles, reimbursement challenges, and proprietary constraints. Increasing the creation and use of explainable models should be a priority for both research and regulatory communities.

### 6.4. Rebalancing Efficiency and Relationship-Centered Care

Building upon these foundational ethical principles, the practical integration of AI into developmental care necessitates a careful rebalancing of efficiency with relationship-centered care. While AI tools promise significant efficiencies, over-reliance could inadvertently erode the crucial relational aspects of developmental care. This section delves into the real-world questions and considerations that arise when striving to uphold ethical guidelines in the implementation of AI.

Based on all of the above, we list the key considerations below:

- Clinical integration and human oversight: AI should augment—not replace—clinical judgment. There must be clinician confirmation and clear referral pathways for assessment and services, with AI acting as a tool for decision support rather than automated labeling. Accountability necessitates human involvement because we do not yet know whether AI can be held to the same degree of responsibility as a clinician or tool.
- Communication and psychosocial support: Receiving a high-risk result can be distressing. Systems must ensure immediate access to counseling, clear next steps, and culturally appropriate explanations, not just a numeric risk score.
- Long-term monitoring and unintended consequences: Continuous post-deployment surveillance is required to detect performance drift, new biases, and downstream social effects (e.g., changes in referral patterns or insurance uses).
- Regulatory, legal and liability issues: It is crucial to clarify whether the tool is a medical device requiring regulatory approval and who is responsible for errors (developers, institutions, or clinicians). Compliance with AI regulations such as the upcoming EU-AI act and health data laws (e.g., HIPAA in the U.S.) is mandatory.
- Resource allocation and access: The introduction of screening tools should not create waitlists or inequitable access to diagnostic follow-up and interventions; otherwise, screening may do more harm than good.

Just as clinicians and families have come to understand and accept the rates of false positives and negatives in established medical tests (like those for gout or Human immunodeficiency virus (HIV)), a similar understanding and acceptance of the probabilistic nature of AI-driven tools will likely develop as they become more familiar

## 7. Future Directions

AI-enabled technologies for early ASD identification are transitioning from research prototypes to clinical tools. To harness their potential responsibly, several priorities must be addressed:

1. Longitudinal validation: Existing studies often report cross-sectional accuracy; future research should evaluate whether early AI-based screening leads to earlier interventions and improved developmental outcomes. Multi-year follow-up of children flagged by AI tools will provide evidence of clinical utility.
2. Dataset diversity and bias mitigation: Models must be trained and evaluated on cohorts representing diverse languages, cultures, ethnicities, socioeconomic statuses, and comorbidities. Standardized reporting of demographic composition and subgroup performance should become normative.
3. Workflow integration and interoperability: Successful implementation requires AI tools that integrate seamlessly with electronic health records, scheduling systems, and referral pathways. Standard data formats (e.g., FHIR) and clear clinical guidelines will minimize disruption to busy practices.
4. Regulatory and reimbursement frameworks: Regulators should continue to develop pathways for evaluating AI-enabled diagnostics, including guidelines for algorithm updates. Payers need to recognize digital biomarkers as reimbursable services to incentivize adoption. Partnerships among clinicians, regulators and industry are crucial to ensure that reimbursement models align with patient benefit, rather than purely commercial interests. It is imperative to establish clear ethical boundaries around data usage, explicitly stating that patient data should not be monetized by private companies. Furthermore, while the benefits of these technologies are significant, it is equally important to emphasize potential risks [13]. These include the climate impact associated with training large AI models and the danger of health systems overlying on automated tools at the expense of investing in the relational and human aspects of developmental-behavioral pediatrics and medicine [13].
5. Ethical stewardship: Developers and clinicians must uphold data privacy, transparent communication, and equitable access. This means ensuring that patient data used by AI tools is collected, stored and utilized with the highest standards of privacy and security, adhering to regulations like HIPAA and GDPR. Transparent communication involves clearly explaining to families how AI tools work, their limitations, and how their child’s data will be used and protected. Equitable access requires actively addressing potential biases in AI models and ensuring that these technologies benefit diverse populations, including those from under-resourced regions or different linguistic backgrounds. Ongoing training will equip clinicians to interpret AI outputs critically and counsel families appropriately, fostering trust and informed decision-making.

## 8. Conclusions

This review highlights that AI-enabled biomarkers—ranging from eye-tracking and voice acoustics to hair metabolomics—have matured significantly, shifting from theoretical research to clinical reality. The systematization of evidence presented here confirms that multimodal AI integration offers superior diagnostic accuracy compared to single-modality approaches. Notably, the recent FDA clearance of devices like EarliPoint and Canvas Dx marks a pivotal shift in the current state of the field, legitimizing digital biomarkers as viable diagnostic aids.

However, while the *technological* capability to detect ASD early and accurately exists, the *systemic* capacity to implement these tools equitably is the next frontier. Current challenges are no longer just about algorithm performance, but about integration into workflows, reimbursement parity, and ensuring training datasets reflect global diversity.

Ultimately, AI does not replace the clinician but elevates them, handling the computational load of pattern recognition so that providers can focus on personalized care. As these technologies scale, they hold the promise of dissolving the diagnostic bottleneck, ensuring that the “wait to fail” model is replaced by a proactive, precision-health approach to neurodevelopment.

## Figures and Tables

**Table 1:** **Summary of AI-enabled tools for identification of Autism Spectrum Disorder.** Each modality represents a distinct data source or signal type—visual (eye tracking), acoustic (speech analysis), behavioral (video or sensor-based), neurobiological (neuroimaging, molecular, metabolomic), or digital (EHR-based and mobile apps). Performance metrics are reported as sensitivity, specificity, accuracy, or area under the receiver operating characteristic curve (AUROC) where available. Reported strengths highlight potential for objective and scalable screening, while limitations emphasize constraints such as data quality, validation across age groups, and generalizability. Tools include both FDA-cleared devices and research prototypes.

Modality	Representative Tool/Data Source	Age Range	Performance Metrics	Strengths	Limitations
**Eye-tracking**	EarliPoint (EarliTec Diagnostics)	16–30 months	Sensitivity 78%, specificity 85.4%, accuracy 82.1% [18]	Objective social-attention measure; 12 min test; FDA cleared	Requires dedicated hardware and brief controlled setting; influenced by visual acuity and attention
**Acoustic/speech analysis**	Voice-acoustic classifier (non-word repetition)	School-age (~8 years)	Accuracy 91% vs. typically developing children, 85% vs. heterogeneous non-autistic group [12]	Non-invasive; low cost; telehealth friendly	Requires verbal ability; small samples; not validated in toddlers
**Video/sensor-based behavior**	Home-video ML classifiers (e.g., ADTree, SVM)	Home videos, mixed ages	Preschool to 6 years Top classifier: 88.9% accuracy, 94.5% sensitivity, 77.4% specificity [10]; specificity 90% for 4–6 y group [10]	Captures gaze and motor features; scalable via smartphone and crowdsourcing	Requires caregiver participation; privacy concerns; video quality affects results
**Neuroimaging**	Research models using Functional Magnetic Resonance Imaging (FMRI), Electroencephalography (EEG)	Preschool to school-age	Classification accuracy ~70–95% in small, homogeneous samples (reviews)	Mechanistic insights; subtype discovery potential	Prohibitive cost; may require sedation; limited real-world applicability
**Molecular/genetic assays**	Gene-expression analyses (blood or saliva)	Perinatal/childhood	Differentiation of genes and networks identified; no stable sensitivity/specificity yet	May reveal biomarkers and targets	Insufficient diagnostic performance: expression varies with stress and environment
**Hair-based metabolomic biomarkers**	ClearStrand-ASD (LinusBio)	Early childhood	Sensitivity ≈ 81%, Negative Predictive Value (NPV) ≈ 92% [19,20]	Non-invasive; dynamic metabolic profiling; high NPV	Not a stand-alone diagnosis [19,20]; results take weeks; limited independent validation
**EHR-based risk prediction**	Predictive ML on well-child data [11] and minimal variables [21]	Birth to 18 months	AUROC 0.83 with 45.1% sensitivity at 95% specificity [11]; AUROC 0.895 with sensitivity 80.5% and specificity 82.9%, external AUROC 0.79 [21]	Uses existing data; scalable; tags risk before overt behavior	Dependent on data quality and access; moderate sensitivity; may miss under-documented cases
**Home-based digital apps**	SenseToKnow (remote tablet app)	17–36 months	Sensitivity 87.8%, specificity 80.8% [22]; Positive Predictive Values (PPV) increases from 40.6% to 63.4% when combined with M-CHAT [23]	Remote administration; can integrate gaze and motor signals; potential	reliance on caregiver compliance, internet access, and the need for training to accurately interpret results, and it has not yet received regulatory clearance.
**Multimodal video + clinician questionnaire + caregiver app**	Canvas Dx (Cogna)	18–72 months	Real-world PPV 94.4%, NPV 95.2%; determinate result rate 60.5% [24]	Combines multiple data sources for enhanced accuracy; FDA authorized	Some cases indeterminate; require caregiver participation; validation across populations ongoing

**Abbreviations:** ASD, Autism Spectrum Disorder; AUROC, area under the receiver operating characteristic curve; EHR, electronic health record; FDA, U.S. Food and Drug Administration; NPV, negative predictive value; PPV, positive predictive value.

## Data Availability

No new data were created or analyzed in this study.

## Abbreviations

The following abbreviations are used in this manuscript:

AI	Artificial Intelligence
ASD	Autism Spectrum Disorder
ML	Machine Learning
NLP	Natural Language Processing
EHR	Electronic Health Record
us FDA	U.S. Food and Drug Administration
NPV	Negative Predictive Value
PPV	Positive Predictive Value
AUROC	Area Under the Receiver Operating Characteristic Curve
CPT	Current Procedural Terminology
CLIA	Clinical Laboratory Improvement Amendments
HIPAA	Health Insurance Portability and Accountability Act
GDPR	General Data Protection Regulation
APA	American Psychological Association
ExAI	Explainable Artificial Intelligence
fMRI	Functional Magnetic Resonance Imaging
EEG	Electroencephalography
MRI	Magnetic Resonance Imaging
rs-fMRI	Resting-State Functional Magnetic Resonance Imaging
rTMS	Repetitive Transcranial Magnetic Stimulation
HIV	Human immunodeficiency virus
FHIR	Fast Healthcare Interoperability Resources
MMPI	Minnesota Multiphasic Personality Inventory
M-CHAT	Modified Checklist for Autism in Toddlers
AAP	American Academy of Pediatrics
US	United States
PCPs	primary care physicians
